# Supplementary figures and images for: Genome-wide identification and evolution of the SAP gene family in sunflower (Helianthus annuus L.) and expression analysis under salt and drought stress
Source: PeerJ. 2024 Jul 30;12:e17808. doi: 10.7717/peerj.17808 (PMC11296301; doi:10.7717/peerj.17808)

zf-A20  
zf-AN1

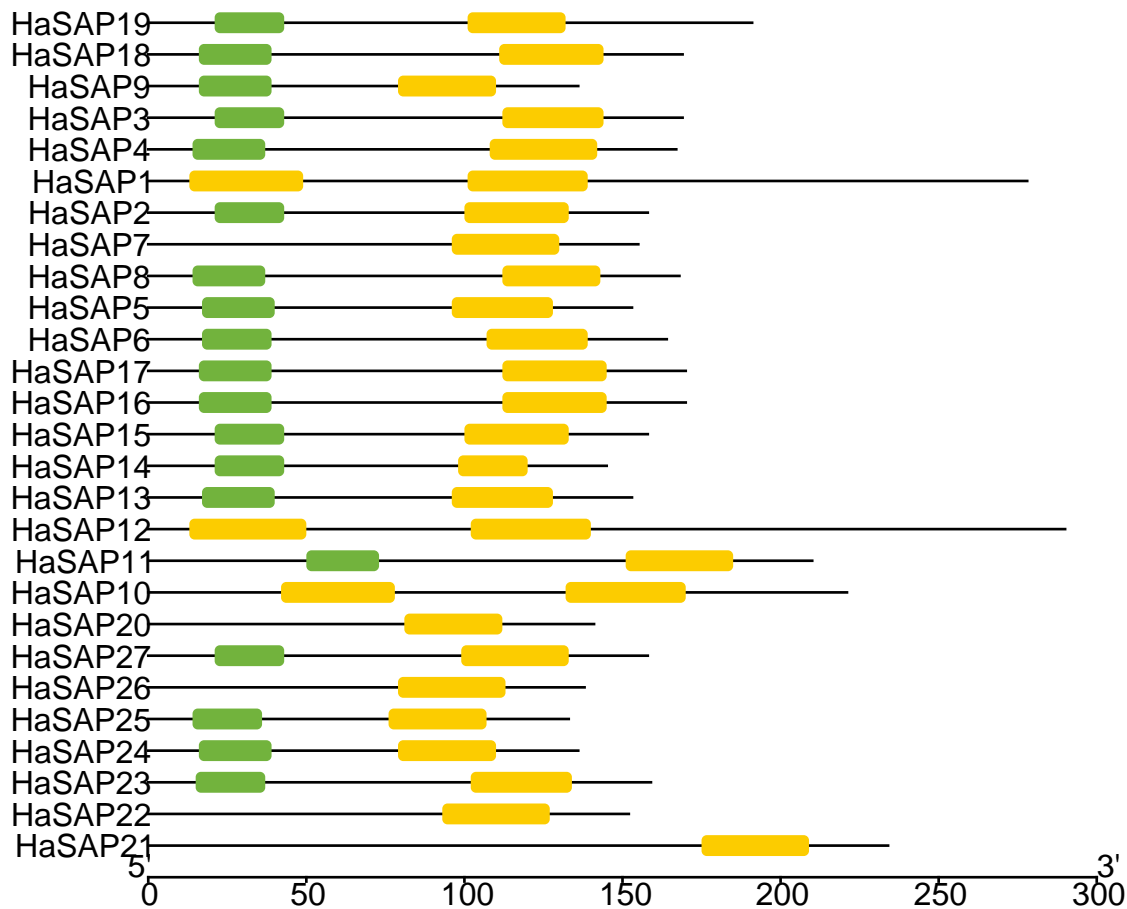

Supplement: Supplemental Information 1 [file peerj-12-17808-s001.pdf]
